# The Physiology of Inflammation and the Healing Process

**Published:** 1844-07

**Authors:** 


### Art. V.

*The Physiology of Inflammation and the Healing Process.* By Benjamin Travers, f.r.s., Surgeon extraordinary to the Queen, &c.—*London,* 1844. 8vo, pp. 226.

Mr. Travers's object, in the unpretending little treatise now before us, "has been to extend our acquaintance with the phenomena of inflammation, as far as the present state of science permits, by the joint aid of physiological and pathological observation, each deriving force from the other."

In proceeding to lay a brief account of it before our readers, we must not fail to express the great pleasure which it gives us, to see a practical surgeon of Mr. Travers's eminence—at a period of life when novelties usually cease to be attractive, and are often regarded with distaste or with positive repugnance and contempt—boldly avowing his conviction, that the pathological doctrines which have been handed down, with but little question, from the time of Hunter, must now be reexamined by the improved light which modern science affords, and tested especially by the facts which the microscope reveals. Even if his volume did not in any way add to our stock of information, we should feel that he well deserved the thanks of the medical world for his good intentions. As a Treatise on Inflammation, we must certainly consider it defective and, in some respects, erroneous; yet, as containing the only account yet published, of the valuable series of preparations made by the late Dr. Todd of Brighton, to illustrate the reparative processes in the frog's web, and as giving to the world the highly interesting results of a series of experiments and observations made by the author himself (with the assistance of Prof. Owen and Mr. John Quekett,) on the same subject,—we believe that it may rank amongst the most important contributions, which the literature of this department has received in later times.

In the Introduction is given a general view of the present state of our knowledge as to the physiology of nutrition, as a proper preliminary to the discussion of the nature of inflammation. In the justice of the following statement we fully concur: "The alterations of which the composition of the blood is susceptible, and those of the relative and reciprocal actions of the blood and the vessels, [or rather, we would say, of the blood and the *tissues*,] which may be fairly presumed, if not ascertained to follow thereupon, are data abundantly sufficient to furnish forth all the phenomena of inflammation, and to supply the rationale of their occurrence." (p. 13.) But we find this statement afterwards complicated by the introduction of the nervous system, as an element in the production of the phenomena of inflammation, to an extent that is not warranted by facts; in this we trace the persistence of certain physiological notions of former years, which, sanctioned by the authority of Hunter, still continue to pervade, more or less, the minds of those who regard themselves as his particular disciples, though inconsistent with the results of wider observation and more correct reasoning:

"It is as indispensable to the understanding of any part of the doctrine of disease, as of the economy of health, to appreciate the influence of this system; all morbid phenomena are traceable to one or other of its properties; and it may be received as an axiom, that the disturbance of consent and relation, as their uniformity turns upon nervous organization, constitutes the most prominent feature, if it be not the exciting cause, of morbid action. Inflammation, therefore, stands in the same relation with all morbid affections, whether functional or organic, to the powers and uses of the nervous organism; nor can we stir one step towards an explanation of its causes, phenomena, and laws, its symptoms or their indications, without appealing to its guidance." (p. 18.)

We are not sure that we understand this extract very clearly; and we regret to say, that a certain degree of obscurity pervades other parts of this introduction. Thus, we are told in the succeeding paragraph, that "Inflammation is susceptible of a purely functional as well as an organic form"—a distinction which, with reference to this department of pathology, we must confess ourselves unable to understand. We observe, too, in p. 11, the following statement, with which we cannot accord:

"The arteries possess a capability of dilatation and a power of recovery; whether muscular, or in virtue of a proper contractility of tissue, or compounded of both, physiologists are divided; Mr. Hunter's experiment showing the difference between the living and the dead artery, notwithstanding. In the larger arteries the muscular, and in the small the contractile property is said to predominate; but whether any order of vessels possesses more than the elasticity which accurately preserves the vessel under its varying dimensions in a state of fulness,is an open question." (p. 11.)

Now, Hunter's statement was, that all the arteries possess *elasticity*, a physical property dependent upon the presence of yellow fibrous tissue in their walls; and also a peculiar vital *contractility*, analogous to that of muscles, and dependent upon a tissue resembling muscular fibre; and, further, that the former predominated in the large arteries, and the latter in the small. Now, as this statement has been fully confirmed by other anatomists and physiologists, who have proved that, in all its essential conditions, the contractility of the arteries resembles that of the lowest forms of muscular fibre,—and as the microscopical inquiries of Henle and others have shown that fibrous structure analogous to the non-striated muscular coat of the alimentary canal exists in arteries, we cannot see any ground for doubting Hunter's conclusions. And we think that in separating *muscularity* and *contractility*—assigning one to the large and the other to the small arteries—Mr. Travers shows a vagueness of conception as to those properties, which we should not have expected from him. He proceeds to say, "that the proof of *elasticity* is furnished by the varied states and rapid changes of the capillary circulation in the temporary phenomena of blushing;" but these we should assign rather to the *muscularity* of the arterial walls, seeing that the changes in question are undoubtedly under the influence of the nervous system, which, so far as we know, has no power over the property of simple elasticity.

In the first chapter are stated the preliminaries or postulates, on which Mr. Travers's view of the character of the inflammatory process is based. These, however, do not so far differ from the ordinary statements on the subject as to require any remark from us. He repeats the assertion, that the nervous system is essentially connected with the process, in the following form:

"Inflammation is not seated exclusively in the blood, the blood-vessels, absorbents, or nerves of the part, nor confined to the capillary system of vessels, nor the cellular tissue which connects and supports them. These several parts forming a homogeneous whole, cannot be disjunctively affected, in morbid more than in healthy action ; each being an integral element in the composition of the other, reciprocating organization and function, acting and acted upon respectively, their peculiar functions are merged in the production of general phenomena, whether healthy or morbid, to which each is indipensable. Thus neither heat, pain, swelling, nor redness could be demonstrated in inflammation, more than temperature, sensation, figure, and colour could be maintained in health, if the blood or the vessel, the nerve or the absorbent were taken away. We might as well expect nutrition to go on in the absence of the stomach." (p. 26.)

Now we think that, in this passage, Mr. Travers confounds the external signs of inflammation, with the essential nature of the process. If it be, as physiologists now seem inclined to regard it, a perverted form of the ordinary action of nutrition, it may be as independent of the nervous system as is the normal process which it represents; and the fact that inflammation may take place in paralysed parts, and even in parts whose nerves are uninjured, without pain, seems a proof that the nervous system is not a necessary and essential participator in the phenomena of inflammation, though usually concerned in the production of the symptoms or manifestations of this process, both local and general.

Mr. Travers takes exception to the doctrine propounded by Dr. Macartney, that the healing process may be effected without inflammation ; and that the latter action, so far from being favorable, is adverse to healing. On this point we shall presently offer a few observations, when noticing another part of the treatise.

The two succeeding chapters, on the direct effects of stimuli and of wound, and on the local symptoms of inflammation, do not offer us anything peculiarly calling for remark. The constitutional symptoms, or effects of inflammation on the system, are next discussed ; and this in a manner, which shows all that power of observing phenomena, and of drawing from them sound practical deductions, which we should anticipate from a surgeon of Mr. Travers's experience. The general state of the system suffering under acute inflammation, and the *irritative* form of the fever accompanying certain injuries, or occurring in bad constitutions, are most graphically described ; and are rightly attributed, we think, partly to the altered condition of the blood, and partly to the affection of the nervous system. That the increased quantity of fibrin in the blood, and other alterations in its quality, must operate as disturbing causes on the general functions of the system, can scarcely, we think, be doubted; but on the other hand, as Dr. Williams justly remarks, the constitutional disturbances does not bear a relation by any means constant to the alteration in the condition of the blood ; and it often seems as if it preceded the local affection. Moreover, we find that the varying excitability of the nervous system in different individuals, has a great influence on the degree of constitutional disturbance produced by a local inflammation ; and it seems to be from the low degree of this in most of the inferior animals, as compared with man, that their system is generally affected much less than his, by severe injuries or local diseases.

The observations on bloodletting as a remedy for inflammation are so valuable, from their being at the same time scientifically true, and practically important, that we shall transfer them almost entire to our pages.

"The choice of measures, *i. e.*, local or general bloodletting, is determined, partly by the relation of the parts affected to the centre of the circulation, and in part by the more or less urgent necessity that exists for disembarrassing the general functions, and arresting destructive inflammation. In visceral inflammation, venesection is [often] indicated and warranted to the utmost extent that the powers of life will bear; for here the mass of blood is so altered and spoiled for its proper and healthy purpose, by the direct implication of the blood-making and blood-preparing organs in the disease, that relieving the system of its presence to the extent that can be borne, is the main resource we possess for its preservation. As we would remove a poison, a *materies morbi*, in such cases we take away blood. .... A freer circulation through the small vessels, and those of the excretory glands especially, ensues almost immediately upon a full bloodletting; the sense of overwhelming oppression is relieved, and the inflammation, if not abridged by its effects, is disposed to a kindlier termination.

"There are two false doctrines concerning bloodletting for inflammation, which cannot be too strongly condemned; the first, anticipatory bloodletting, by which I mean, the large and repeated detraction of blood, before inflammation, being considered inevitable, has actually manifested itself, on the hypothesis of starving the action, and thus rendering it tractable, which is a direct attack on the vitality and fatally perverts the action, if it do not destroy the resisting powers of the system. The second, continuing the employment of the lancet, so long as the last-drawn blood exhibits the signs of inflammation, which if drained to the last drop it would do; or, in other words, not reflecting that there is a line beyond which the practice becomes destructive instead of remedial; and that there are many inflammations which do not admit of arrest by depletion, and upon which other modes of treatment are efficient for this end, even though not an ounce of blood be drawn. Many lives have been sacrificed to the prevalence of these irrational and absurd notions; and many preserved in their extremity by being fortunately placed beyond the reach of the surgeon; especially, I am induced to believe, in military practice.

"When the change of the mass of blood ensues upon local inflammation, whereever seated, some of the internal organs especially, but all more or less, indicate the effect produced by it, and fever is established. .... It may be asked why, if this reasoning be correct, we do not combat the general inflammatory fever of the system, when arising from a complicated injury, or fracture, or ulcer of the extremity, as when affecting the visceral organs? the answer is clearly this, that in the former case the inflammatory fever is secondary, and not idiopathic; that the local injury or inflammation attending it has already produced its effect upon the nervous system, to the extent of materially deranging and depressing its powers; and that we dare not reduce them directly, as by the lancet, lest we destroy the vitality of the inflamed part, if not sink the general power too low for sustaining life under its burthen. But even in this case, if from a seeming metastasis, or from any cause, the visceral organs indicate inflammation, we do not hesitate to employ general bloodletting, and in all such cases instantly and freely employ topical bleeding." (pp. 58-60.)

The various results of the inflammatory process are next discussed. The effusion of the serum of the blood, which is the first of these, seems to be, in Mr. Travers's opinion, as in ours, a simple physical result of the over-distention of the blood-vessels, and of stagnation of blood in them; taking place, as it does, in so great a variety of conditions, in which inflammation does not at all participate. The exudation of fibrin, however, dissolved in the serous fluid, in other words, of *liquor sanguinis*, is considered by Mr. Travers, as a pathognomonic sign of the presence of inflammation; being effected by a vital action analogous to secretion. "I

cannot view this phenomenon," he says, (p. 74,) "as some have done, as a mere mechanical effect produced by a gorged state of the vessels; if it were, there would be no such uniformity as exists in the results of inflammation; we should oftener have blood poured out *en masse,* and not its separable ingredients; the change commencing within the vessels, and perfected externally. The process is peculiar to life, and the operation of physical and chemical forces is in subservience to vital laws." We are not sure, however, that it is necessary to have recourse to this explanation; and if we *can* fairly explain the phenomenon on physical principles, it is obviously better to do so. The increased tendency to separation between the two elements of the circulating blood, the augmentation in the quantity of the fibrin,—the distention of the vessels, and consequent weakening and thinning of their walls,—the stagnation in the movement, and consequently increased pressure,—would all seem to favour the effusion of the liquor sanguinis; and as it has been clearly proved by the experiments of Mr. Robinson, that this *may* escape from the vessels without inflammation, but under physical conditions which resemble those produced by the inflammatory process, we are much inclined to refer the effusion of fibrin in an inflamed part, to the same causes. That it should take place so constantly, is only to be expected from the uniformity of the preliminary changes; and there could be no effusion of blood *en masse,* without an absolute rupture of the vessels. We would not wish to assert dogmatically, however, that no process resembling that of secretion, is concerned in the effusion of fibrin from inflamed vessels; but merely desire to point out that physical causes, known to be in operation, will go a great way to account for it.

The process by which the organizable fibrin, poured out into a wound with loss of substance for the purpose of reparation, forms a vascular communication with the surrounding parts, is extremely well described; and evidently from the author's own observations. We should gladly have transferred the whole to our own pages, did our limits permit; but we must be content with quoting the following summary:

"Every wound presents the following phenomena: the occlusion of all divided vessels, not of large caliber, by clot or coagulum of blood; the actual stasis of the circulation in the part, the first local phenomenon of inflammation, with or without lesion; the gradual deposit of fibrin devoid of colour, and the subsequent more gradual vascularization of that fibrin, by the opening of transparent channels for the admission of at first isolated globules, then a train or file of globules, and ultimately a column conveying colour; their inter-communication, anastomosis and reflection towards the part whence they are derived; and lastly, the offset of stagnant meshes, like advanced posts from the parent blood-vessels, slowly acquiring motion and dispersing. All tend to demonstrate that the laying down of the organizable fibrin, the intrusion of the blood-corpuscle, and thence the formation of the new vessels, are the work of active coloured capillaries nearest to the point of stases, aided and completed by the return of circulation, also gradual to the vessels, trunk and branch, which had been rendered motionless by congestion." (p. 80.)

Mr. Travers does not admit that there is such a thing as isolated or independent vascularization, —i. e., the production of vessels in the organizing lymph, not originating in the subjacent vascular surface; and he gives a different explanation of the appearances, which have induced some pathologists to admit such an inference. He states, however, that the vessels are visible in fine striæ, before circulation can be detected in them; and that isolated globules often enter the capillary tube and perform an oscillatory motion for many hours, before any series of them passes into it. Hence we cannot regard the new channel as burrowed out by a string or file of red corpuscles, pushed out from the nearest capillary, by a *vis a tergo*, as some have maintained that it is. Mr. Travers denies that blood effused from wounded surface ever becomes the medium of organized adhesion; and specially refers to Mr. Dalrymple's case, (Medico-Chirurgical Transactions, vol. xxiii,) of the supposed vascularization of a clot of blood effused between the periosteum and bone, as misunderstood by the describer. From our own inspection, however, of a portion of this clot, the vessels in which had been injected from the subjacent surface, we feel disposed to affirm almost positively, that these vessels *could not* have been, as Mr. Travers supposes, those of the connecting cellular tissue of the bone and periosteum, being entirely different in their aspect and distribution. The question, however, is not one of much practical importance; since all are agreed, that blood is much less readily penetrated by vessels, than organizable lymph. We fully accord also with Mr. Travers, in assigning to the process of *rapid* organization, which is seen especially in the ill-elaborated lymph of cachectic subjects, the character of want of permanency;—"slow and sure being a pretty constant couple throughout nature;" and we shall presently apply the same view to some other forms of the healing process. We should have liked to see, in this chapter, the result of Mr. Travers's observations upon the *organization* of lymph or liquor sanguinis; by which we understand the development of cells, fibres, &c., in its substance,—a process which is anterior to its vascularization, and independent of it. The importance of the adhesive process in the economy of nature, and the results of its deficiency in disordered states of the system, are well displayed.

In chapter vii, is an account of the preparations made by the late Dr. Todd of Brighton, to illustrate the successive stages of the healing process, in daily stages, from the infliction of injury to the completion of repair.

"To accomplish this purpose, a corresponding injury was inflicted upon the webs of a large collection of fresh frogs at the same time; and a wounded web being removed at successive intervals of twenty-four hours, was dried, and then placed between two squares of glass, and hermetically closed by a balsamic composition, which preserves appearances even to the shade of colour with remarkable fidelity; the date of each preparation being registered, and carefully engraven on the glass. This interesting collection was purchased of Dr. Todd's executors, by the Royal College of Surgeons, and is now arranged in their museum. The instant appearance of the part, especially of the blood-vessels and their colour, can only be preserved by such a mode of death, as is instantaneous and without struggle, for which purpose Dr. Todd employed nicotine. It is deeply to be regretted that the specimens are unaccompanied with any manuscript record or notes by way of illustration, although subjected to careful examination by the doctor, from time to time, with the design of working out the subject, and ultimately presenting it in a finished state to the profession. Neither the eye nor the mind of a survivor, however accomplished, can supply the place of those of an original inquirer. The time occupied for the completion of the process varies according to the nature of the injury; and allowances must be made for the incidental varieties of vigour or languor in individuals, and perhaps the influence of season, to reconcile the seeming discrepancies in the rate of advance; wherefore an interval of three days furnishes a safer average index of changes, than a shorter period. With such an admission, it is remarkable how striking a uniformity prevails throughout this interesting collection, and how additionally corroborative of general results are these natural and necessary inequalities of progress." (pp. 93-4.)

For the details of these appearances, as presented at successive intervals, after the following injuries,—incised wound traversing an artery, incised wound traversing a vein, incised wound in the direction of the toes, excision of a circular portion of the web by a punch, contusion, and burn,—as well as for a brief summary of the appearances exhibited by each series,—we must refer our readers to Mr. Travers's work. We may state, however, that the reparative processes appeared to be of the same character, in all instances, with those which are seen in the first sprouting of the feet of these animals; as soon, at least, as the immediate effect of the injury was recovered from.

The formation of granulations, and the process of suppuration, treated of in chapter VIII, are regarded by Mr. Travers, as constituting "the second or advanced stage of adhesive inflammation, providing repair not only for solution of continuity, but for loss of substance." We formerly expressed our concurrence in the opinion of Dr. Macartney, that granulation and suppuration are not by any means essential to the closure of a wound or ulcer, in which there is great loss of substance; and that they are to be avoided if possible, both on account of the constitutional disturbance they engender, and the imperfect nature of the local restoration,—the endeavours of the surgeon being rather directed towards the establishment of a process resembling that of natural growth, which, though *slower*, is much *surer*. For in the latter, the new tissues resemble those which they have replaced; whilst granulations consist of an entirely different structure, very rapidly organized, but of low vitality, and not permanent in their character; so that they are absorbed at a subsequent time, and produce a contracted cicatrix. We are further inclined to regard the effusion of liquor sanguinis in the degenerated or degraded form of pus, as the consequence of the low vitality of the part from which it is exuded; a view which corresponds very well with the known action of the influences which produce suppuration. We know that these views will not spread rapidly amongst our older brethren, who have been accustomed to regard suppuration as a *healthy* process, when the wound is in a healing state; but we would ask whether any reparation can be more complete than that which takes place in the frog tribe,—new legs, with perfect bones, muscles, nerves, &c., being often produced after the severe loss or injury of the original ones,—whilst no pus-globule, nor any fluid resembling pus, can ever be detected during the process?

We find, in a later part of the work, some further observations upon the constitution and origin of pus; from which it appears that Mr. Travers regards this fluid as consisting of the "superfluous or waste lymph, which has been separated during the adhesive stage, from the mass of the blood, held in solution by the serum, being thus a chemical modification of the constituents of the liquor sanguinis; in short, the latter fluid deprived of its original characters and power of spontaneous coagulation." (p. 172.) We do not see that the mere dilution of the fibrin with serum, can destroy its coagulability; since we know that a very minute quantity of fibrin diffused though a serous fluid, will occasion the formation of a coagulum, however slight. We are much more inclined to regard the aplastic character of pus as the result of a change in the *vital* endowments of the fibrin of the liquor sanguinis at the time of its exudation. The effusion of pus upon the surface of granulations is considered by Mr. Travers as necessary to their maintenance, affording them a bland and homogeneous protecting coat; this may be very true,--but the question still is, whether it is desirable to encourage the process, and whether the closure of a wound may not be often effected, under seclusion from air, and a regulated temperature, by a process of a much more satisfactory kind than granulation and suppuration. We think it can; and that the " slow and sure" operation to which we have adverted, is worth more attention than Mr. Travers has given to it.

In order to complete the history of the healing process, which the cabinet of Dr. Todd partially displayed, Mr. Travers carried on, with the assistance of Mr. John Quekett, a series of observations on the effects of injuries of the frog's web,—adopting the more useful plan of permitting the animals to live, so that the stages of the healing process might be observed and recorded, until it was perfected in the same individuals. The ninth chapter of Mr. Travers's work, which contains his account of these observations, we regard as the most valuable in the whole work; and we only regret that it was not expanded into a separate memoir, and accompanied with such delineations as, without being too elaborate in their execution, might have conveyed a better idea of the successive stages of the process than any words can do. We are sure that our readers will thank us for placing before them the following recapitulation or digest of the phenomena observed during the early period of reparation. The return of the circulation has been already noticed:

" 1. Stasis, or actual arrest of the circulation, is a direct effect of local irritation, more or less persistent, according to the degree or shock. Thus, if unattended by injury to structure, recovery from it restores the previous condition, whether inflammation be set up or not. Its local extent, like its duration, will be according to the amount of the irritation. The circulation is oscillatory at the verge of the stasis; beyond this it is preternaturally slow; and yet further from the stationary centre it is, or appears to be, somewhat brisker than natural. The suddenness and completeness of the stasis determine the acuteness of the inflammation; it is slowly formed and imperfect in what are termed congestive inflammations.

" 2. Contingent upon the stasis is the effusion of serum or of the liquor sanguinis, the one or the other, according to the nature of the injury inflicted, whether of function, simply, or of structure. If serum only be effused, the inflammation, if any be present, admits of perfect resolution; not so, if the effusion be of the liquor sanguinis holding the fibrin of the blood in solution. This is the distinction between the effusions of shock and of lesion, the nervous and vascular, the inflammatory and non-inflammatory œdema. The second results from a more considerable and prolonged action than the first, if both be the effects of inflammation; but whereas the aqueous effusion is often unattended with inflammation, the fibrinous effusion is characteristic and proper to it, whether with or without primary breach of texture.

" 3. The fibrin effused in a state of solution in the liquor sanguinis only becomes susceptible of organization; i. e., capable of permanent incorporation with the living solids, when separated from the other constituents of the blood......

"4. The effusion of the liquor sanguinis from the arterial capillaries is the first change consequent upon inflammation, either with or without breach; it is, in either case, limited to the extreme verge of vascular action, or the boundary-line of its arrest (complete stasis). In the act of coagulation upon the face and sides of the wound, the contained fibrin separates from the serous portion of the liquor sanguinis, and becomes a crust or membranaceous stratum, covering in the wound at all points, the sections of blood-vessels, nerves, absorbents, &c. This, then, forms the intermedium of vascular communication by anastamosis, in cases of union by adhesion..........

"5. The second act in the healing process is the separation of the lymph-particle from the blood within the vessel. This is not seen until after some time has elapsed, for it is not an immediate consequence of the stasis, which may and does continually happen for short and definite periods, without this separation or any effusion of lymph, as in simple irritation or inflammation of a sound texture without deposit. The necessity created by extensive breach, or injury determining such breach, establishes the adhesive action on a scale of extent and duration proportioned to the loss incurred by the injury; and the effusion—always marginal only—during the period of stasis, and maintained so much in advance of the active circulation, as almost to convey the idea of its being derived from some other source, is now in the form of lymph-globule, which has become separated within the arterial coloured capillary, for the special purpose, as it would seem, of supplying the permanent plasma or new solid. Its appearance in the veins is not recognized until the stasis is passed and the circulation restored. Neither is it seen in the newly-formed vessels, but only in the vessels which have undergone the stasis. These are preternaturally dilated; the venous side of the capillary circulation appears to be that most loaded; it is at least most conspicuous. The restored circulation of the part is slow and laboured; the deposit is not only limited to the verge of the wound, but its progress and the rate of healing bear a steady reciprocal relation to each other.

"The elaboration of organizable lymph appears to become perfected as it advances, confirming the practical observation, that wounds which heal slow heal sound. It is quite a mistake to suppose that, in wounds attended with loss of substance, the effusion of organizable fibrin constitutes the permanent material of the new solid. The first deposit effused with the liquor sanguinis is an amorphous exudation, and presents no such regular figure and arrangement as the lymph-particle which has been separated within the vessel before deposit. They are, in truth, different in this respect; the first, or that which forms immediately on the receipt of injury, and serves for the intermedium of organization in a close apposition of surfaces, (being separated from the liquor sanguinis, with which it is effused,) would not serve as a base for the new solid; it is soon absorbed, being only a temporary bond or adhesive layer, in harmony with the parts, though serving the important purpose of consolidation by anastamosis of the contiguous vessels of the opposite sides, or union by the first intention; the second is not called for in mere divisions of substance, and is not ready if it were; it requires a higher and long-continued inflammatory action; it is a permanent, not a provisional mean of reparation—a substantial addition of structure, not a mere conjunction of parts." (pp. 160-6.)

There is a little obscurity in the above statement, as regards the separation of the lymph-corpuscles; but, on looking into the detailed accounts of the observations, we find the following passage, descriptive of the appearance of the web on the twentieth day after the injury, which fully explains the author's meaning:

"Active circulation in new vessels, to the outer margin of the lymph-layer bordering the wound; a very great number of the isolated round particles of lymph in the circulating vessels, not forming part of the current, nor blended with the blood, but stationary, either single or in groups, then carried on from time to time in vessels which have the appearance of veins; again, these or similar particles accumulated at the outer edge of the wound permanently at rest, and disposed in rows, forming the new lymph-bed of organization." (p. 145.)

From these observations, Mr. Travers has been led to an inference respecting the function of the colourless or lymph corpuscles, which closely corresponds with that upheld by Dr. Carpenter in his Report on the Physiology of Cells,* and which affords a striking confirmation of it; viz., that they are concerned in the elaboration of the organizable lymph or plasma, at the expense of which the new tissues are produced. It would seem, from Mr. Travers's observations, as if the material first effused were the ordinary liquor sanguinis of the blood, and as if this were not sufficiently organizable to become an entirely *new and permanent* tissue, though adequate both to afford nutrition to the old, and to form a new tissue of temporary character. It is not, according to him, until this increased development of lymph-corpuscles manifests itself in the vessels of the part, that the permanent new tissue is generated.

Now it is a very interesting question, whether the healing process, as thus described, is to be regarded as of an inflammatory character or not. We think not; and for these reasons: The process is exactly analogous to that which is witnessed in the first development of many new parts during embryonic life; as, for example, the crystalline lens, and the extremities when they begin to bud out from the trunk. In both of these cases there is a retardation of blood in the neighbouring vessels, which are much dilated, and a great accumulation and stagnation of the colourless corpuscles, which appear to elaborate the organizable matter required for the new growth. All these, therefore, are merely acts of formation taking place with unusual activity. A similar condition is seen in the vessels of the Fallopian tube during the passage of the ovum, around which the chorion is formed by the exudation from the lining of the tube; and the same would probably be found in the spots at which the rapid growth of the stag's horns takes place, or any other act of new formation, to which no one has ever thought of applying the term inflammation. In all these cases, there is no change in the general character of the blood; because the action is one of a purely local kind; and as fast as the new fibrin is generated, it is used up in the formative process. But if this production were *too* rapid, there would then be an accumulation of fibrin in the blood, and a part of it would probably pass off in the unorganizable or aplastic form of pus. We reserve, however, the detailed discussion of this subject for another article; and shall now conclude with a few words on the closing chapters of the work before us, which treat of ulceration, cicatrization, and gangrene.

Mr. Travers is a staunch advocate of the Hunterian doctrine, that the absorbents are the vessels chiefly, if not solely, concerned in the removal of the tissues by ulceration; and he combats at some length the objections of those, who have maintained that the veins are the agents of this operation. His arguments, however, are chiefly based on the assumption, (for such we must term it,) that the debris left by the ordinary waste of the system are taken up by the lymphatic vessels; a doctrine which has been lately called in question, and of which there does not

* Brit. and For. Med. Rev., Jan. 1843.

seem any sufficient proof. The question seems to us, however, of very little practical importance ; that of the condition of the parts disposing to ulceration is of much greater consequence. It is not, in our apprehension, an increased action of the absorbent vessels (whether veins or lymphatics,) which is the primary cause ; but an increased waste or disintegration of the tissues, resulting from previous changes in their state. There is, in fact, as it seems to us, a molecular death of a certain amount of tissue, whose debris are removed by the absorbent vessels; the difference between ulceration and sloughing being simply this,—that in the latter, the whole substance dies at once, and separates from the living part, so as to be thrown off *en masse ;* whilst in the former, the death is more gradual, and the process of interstitial absorption still continues active, so that the products of disintegration are removed as fast as they are set free. The latter is consistent only with a state of parts, in which the vitality has been less completely destroyed than in the former process; and, consequently, we find ulceration taking place at the edge of a slough.

We shall only further say, that these closing chapters, concise as they are, abound with important practical observations and deductions ; and that the whole work, though certainly far from being a complete treatise on the subject, may be strongly recommended to the student and practitioner, as well as to the professed pathologist. On the latter, Mr. Travers will confer a great boon if he will follow out his series of observations on a more extensive scale, and give their results in a more extended and elaborate form.

---

